# Supplementary material for: Sequence variants affecting the genome-wide rate of germline microsatellite mutations
Source: Nat Commun. 2023 Jun 29;14:3855. doi: 10.1038/s41467-023-39547-6 (PMC10310707; doi:10.1038/s41467-023-39547-6)
Supplement: Supplementary file 1 — Supplementary Information [file 41467_2023_39547_MOESM1_ESM.pdf]

Supplementary information

Supplementary note 1: STR properties correlate with polymorphism rate and expected heterozygosity

A number of STR properties correlate with the polymorphism rate and expected heterozygosity[1]. Both the motif length and how many times the motif is repeated in its RRT (repeat number) affect the polymorphism rate and the expected heterozygosity. Almost all homopolymers are polymorphic and both the fraction of polymorphic STRs and expected heterozygosity decrease with motif length (linear regression $P < 1 \cdot 10^{-320}$ for both datasets, Supplementary Figure 1, Supplementary Figure 2). For all motif lengths, an increase in repeat number results in both a higher polymorphism rate and expected heterozygosity (linear regression $P < 1 \cdot 10^{-320}$ for both datasets, Supplementary Figure 1, Supplementary Figure 2).

The fraction of G/C bases in an STR‘s motif (motif GC content) is negatively correlated with polymorphism rate (Supplementary Table 5) while negative correlation with expected heterozygosity is only observed for motif lengths above two bp (Supplementary Table 4). Homopolymers from the C motif class have higher expected heterozygosity than A class homopolymers (Supplementary Table 4) but account for only 0.8% of all homopolymers (Supplementary Table 24). CpG microsatellites (CG motif class) also have on average higher expected heterozygosity than the other dinucleotide motif classes but account for only 0.4% of all dinucleotide repeats (Supplementary Table 24, Supplementary Table 4). Thus, although C class homopolymers and CG class dinucleotide microsatellites have higher expected heterozygosity values than other classes with equal motif lengths, their overall effect on microsatellite diversity is small since they are so rare.

Enrichment of A motif class homopolymers in the human genome is thought to be a result of the microsatellite-like structure often found at 3‘ ends of reverse transcribed RNA sequences, i.e. poly-A tails[2,3] and the high expected heterozygosity rate at CG class microsatellites is consistent with CpG sites acting as mutational hot spots[4]. Negative correlation of GC content to polymorphism rates and expected heterozygosity can likely be explained by the three hydrogen

bonds between paired G/C bp, compared to two between A/T bp, making the slippage-causing disassociation from the template strand during replication less likely.

We define repeat purity as the ratio between the number of times the STR's repeat motif is observed in its RRT and the maximum number of repeat motifs if the sequence contained no interruptions (where the highest possible repeat purity is 1). Stratified on RRT length, all correlations between repeat purity and both expected heterozygosity and polymorphism rate were positive (Supplementary Table 2, Supplementary Table 3, Supplementary Figure 6, Supplementary Figure 7). This is consistent with[5–8], reporting a positive correlation between repeat purity and mDNM rate. Interruptions of the repeat sequence decrease the number of locations where replication slippage can occur and thus the mDNM rate[9].

Supplementary note 2: False positive rate estimation

To estimate the false positive rate of our mDNM detection we used three methods; PacBio CCS sequence data, available for four of our trios, mDNM sharing between nine monozygotic twin pairs and haplotype sharing across three-generation families We used haplotype resolved assemblies of the PacBio data and while we were unable to verify homopolymer mDNMs as the PacBio sequencing error rate was too high[10] we were able to verify the existence of 27 mDNMs with motif length >1. Out of these, 26 were true positives and one was a false positive at a dinucleotide repeat, giving an expected false positive rate of 3.7% for motif lengths greater than 1. (Supplementary Table 7).

For mDNMs observed in offspring with a monozygotic twin also present in our set, we checked whether the mDNM genotypes were concordant between both twins. We compared mDNM calls where the genotype was present in the monozygotic twin of the offspring. We treated genotype calls of the other twin present if the genotype quality was higher than or equal to 30, which is half of the value we require for trio mDNM detection. Out of the 230 comparable MZ-twin mDNMs, 217 were found in both twins and 13 were discordant (Supplementary Table 8) which gives a false positive rate estimate of 5.6%. We note that this is likely to be an overestimate, as some of the differences between the twin pairs could be due to result of post zygotic mutations, representing true differences between twins[11].

Using haplotype sharing across 540 three-generation families (795 trios), we counted how many times an mDNM was transmitted from an offspring to its child and estimated the transmission rate. The expected value of the transmission rate is 0.50 and deviations from it quantify false positive mDNM detection rates. For example, if the observed mutations were somatic and thus false positive as mDNMs, we would not observe transmission from the offspring to its child. We observe a transmission rate of 0.49 (N = 11,228, 95% CI: 0.48-0.50) which gives an estimated false positive rate of 2%, although transmission rates vary between motif lengths and thus the error rate estimates as well (Supplementary Table 9). Notably, the transmission rate for homopolymers is only 0.4 while the other motif lengths have transmission rates much closer or equal to 0.5.

Supplementary note 3: mDNM rate estimate comparison

Our mDNM rate estimate is nominally lower than a previous estimate[12] of $5.6 \cdot 10^{-5}$ and lower than the two estimates of $10.0 \cdot 10^{-4}$ and $2.7 \cdot 10^{-4}$ for tetra- and dinucleotide repeats, respectively[13]. This apparent discrepancy could be a result of a more conservative filtering in our study, a younger set of parents, a generally healthier cohort and a different range of motif lengths considered. However, the most likely reason for the apparent discrepancy is the sample size difference between the studies. Our set contains 53,026 individuals while the set analyzed by Mitra et al. [12] contained 6,548 individuals. Thus, our minimum detection frequency is $1/(2 \cdot 53{,}026) = 9.0 \cdot 10^{-6}$ compared to the minimum detection frequency[12] of $1/(2 \cdot 6{,}548) = 7.6 \cdot 10^{-5}$ enforced by the smaller sample size. We recomputed our mutation rate estimate conditioning on microsatellite frequency (Supplementary Table 25) and confirmed that at a detection frequency cutoff of $7.6 \cdot 10^{-5}$ our estimate becomes $5.6 \cdot 10^{-5}$ and matches the one presented by Mitra et al.[12]. Similarly, at a minimum frequency of 10% our estimate is comparable to the one from Sun et al.[13] Based on this we conclude that a mDNM rate estimate depends on the size of the sample set studied.

Supplementary note 4: mDNM rate comparison between motif equivalence classes

The mDNM rate is higher for C class homopolymers than for A class ones (Mann-Whitney U test $P < 1 \cdot 10^{-230}$, Fig. 2), but C homopolymers are much rarer and represent only 0.8% of all homopolymers in our set (Supplementary Table 24). The AC motif class has the highest mDNM rate of the dinucleotide microsatellites (Supplementary Table 26, Fig. 2). However, the average RRT length of the AC motif class is longest among dinucleotide classes. Including RRT length as a covariate the CG motif class has a higher mDNM rate than all other dinucleotide classes (Supplementary Table 27), in line with the fact that CpG sites have been shown to act as mutational hot spots[4].

The AAT motif class had a higher mDNM rate than eight of the other nine trinucleotide repeat motif classes. Only the rarest class (ACG) did not show a significantly different mDNM rate (Supplementary Table 28). The AAT motif class accounts for 38.4% of all trinucleotide microsatellites and 79.0% of trinucleotide mDNMs and has an mDNM rate 1.5 times higher than the second highest class (Supplementary Table 28). A higher mDNM rate for AAT class motifs has been previously reported for other organisms[14,15] but not, to our knowledge for, humans.

An in vitro study of how repeat motifs affect the frequency of polymerase slippage during replication reported that motifs less likely to stall replication were more likely to mutate during replication. Of the dinucleotide repeat classes, microsatellites from the AC class had the lowest replication stall affinity[16]. The higher mDNM rate of microsatellites in the AAT motif class could be a result of its low replication stall affinity[16]. The two hydrogen bonds between A/T base-pairs compared to the three between G/C base-pairs also makes A/T pairs more likely to disassociate from each other, enabling the formation of secondary structures and possible mDNMs. Finally, repeats with a high A/T-content also have a sequence composition similar to elements involved in DNA unwinding at replication origins[17] during mitosis. These repeats could therefore function as aberrant replication origins and cause a higher mDNM rate during replication in S phase[17].

Supplementary note 5: mDNMs in functionally annotated and early replicating regions

Previous studies have reported increased efficiency of mismatch repair (MMR) in early-replicating regions of the human genome[18]. Our results are in line with this since we see 1.28 (95% CI: 1.25-1.31) fold depletion of mDNMs in early replicating regions of the genome[19].

Exonic mDNMs are rarer than their intergenic and intronic counterparts. In 2,568,858 transmissions of microsatellites intersecting exons by one or more bp, we observed 33 mDNMs.

We estimated the exonic mDNM rate as $1.3 \cdot 10^{-5}$ MMG, which is 3.9 (95% CI: 2.8-5.6) times lower than the genome-wide estimate. mDNMs are further 1.7 (95% CI: 1.3-2.1), 1.4 (95% CI: 1.3-1.5) and 4.2 (95% CI: 1.2-34.4)-fold depleted in 5‘UTR and 3‘UTR and splice regions, respectively. The 33 exonic mDNMs occurred at 21 unique microsatellites, of which 19 had motif lengths that were multiples of three and since amino acids are coded with three bp codons, mutations at microsatellites with multiple of three motif lengths are unlikely to cause a frameshift but rather an in-frame alteration of a gene. Sixteen of the exonic mDNMs were trinucleotide microsatellites, three were hexanucleotide microsatellites and the remaining two were homopolymers.

Tri- and hexanucleotide repeats were enriched in coding exons (chi squared test $P < 1 \cdot 10^{-320}$) compared with the rest of genome. Microsatellites with motif lengths that are multiples of three accounted for 93.3% of exon intersecting microsatellites and 70.9% of all exon intersecting non-polymorphic STRs (Supplementary Table 29). In contrast, 12.3% of all microsatellites had motifs that are multiples of three (Supplementary Table 29) and 44.5% of non-polymorphic STRs.

The average purity of microsatellites was 0.94, while among microsatellites in exons the purity was notably lower (0.87, Mann-Whitney $P = 1 \cdot 10^{-153}$). Purity was positively correlated with the mDNM rate, so decreased purity in exons may decrease occurrences of possibly pathogenic mDNMs. This indicates that there is a possible positive selection for point mutations that reduce the purity of exonic microsatellites or a possible negative selection for point mutations that increase in their purity. The purity difference is largest for trinucleotide repeats, the most common motif length for exon intersecting microsatellites (Supplementary Table 30). Non-polymorphic coding STRs do not have decreased purity compared to their intergenic

counterparts, so point mutations are less likely to be the mechanism preventing mutations at these exonic STRs (Supplementary Table 30).

Supplementary note 6: Replication of mutation rate trends

As the RRT length increased from ten to 100 bp the mDNM rate also increased for all motif lengths (Fig. 2), consistent with findings from previous studies[20–23] and, intuitively, the opportunities for errors during replication increase with a microsatellite's RRT length.

Microsatellites with longer RRTs have been shown to be more likely to contract and shorter ones to expand[23–29]. We replicate this. For all RRT length thresholds, a higher fraction of mDNMs had a gain of repeat motifs below the threshold than above it, i.e., the microsatellite group with shorter overall RRTs were more likely to expand than the group with longer RRTs (Supplementary Table 31).

The mDNM rate for homopolymers was positively correlated with the motif G/C content, while di-, tri-, tetra- and pentanucleotide repeats had a negative correlation with the mDNM rate and for hexanucleotide repeats we lacked power to detect a correlation with the mDNM rate (Fig. 2, Supplementary Table 22).

Repeat purity correlated positively with the mDNM rate for all motif lengths (effect = 0.12, $P < 1 \cdot 10^{-320}$, Fig. 2, Supplementary Table 22), consistent with previously published results[22]. The correlation remained positive in most cases after conditioning on RRT lengths (Supplementary Table 32).

mDNM rates varied by repeat motif class, AAT motif mDNMs were by far the most common ones among trinucleotides (Supplementary Table 28). The rare C homopolymers mutated more frequently than the A homopolymers and AC motif microsatellites had the highest mutation rate of the dinucleotide microsatellites before correcting for RRT (Supplementary Table 26) length and CG motif microsatellites after correction (Fig. 2, Supplementary Table 27).

Supplementary note 7: Motif length enrichment excluding homopolymers

A higher fraction of maternal mDNMs occur at tri-, penta- and hexanucleotide microsatellites while dinucleotide microsatellites represent a larger fraction of paternal mDNMs (Supplementary Table 14, Fig. 3).

The average number of bp involved without homopolymers is larger in maternal mDNMs than in paternal mDNMs (3.9 vs 3.4 bp, Mann-Whitney U test $P = 2.6 \cdot 10^{-23}$). Stratifying on motif length reveals that maternal mDNMs affect more bp on average at di- and tetranucleotide microsatellites (Table 2).

Considering mDNMs with motif lengths above one, the number of repeats in the reference is higher for paternal mDNMs (16.8 vs 15.7 repeats, Mann-Whitney U test $P = 2.8 \cdot 10^{-51}$).

Supplementary note 8: Motif length fraction change with age including homopolymers

Tetranucleotide mDNMs increase their fraction with paternal age (Linear regression $P = 1.5 \cdot 10^{-6}$) and the fraction of di- and hexanucleotide mDNMs increases with maternal age.

For both maternal and paternal mDNMs the fraction of mDNMs at homopolymers decreases with age.

Supplementary note 9: Mismatch repair efficiency effect of rs4987188

Studies of G317D, the yeast homolog of rs4987188, conclude that it does not affect *MSH2* expression levels, rather that protein products of the mutated allele have a decreased mismatch repair efficiency relative to the wild-type allele and need to be expressed at higher levels to be equivalent to it[30,31]. The first experiment was a direct comparison of mismatch repair rates for the wild-type allele and G317D, resulting in a significant 1.7 MMR defect. The second experiment compared how well G317D could complement a msh2Δ-null mutant when expressed from the native *MSH2* promoter and when expressed at higher levels from a GAL10 promoter. When expressed from the GAL10 promoter, the yeast G317D allele partially complemented the msh2Δ-null mutant, in turn no complementation was observed when it was expressed from the native *MSH2* promoter. Combined, these results suggest that G317D affects MMR efficiency and that in vivo, the function of this variant could change with levels of

expression. To emphasize how this supports our results, we generated a boxplot of the RNA-expression of *MSH2* for each rs4987188 genotype, (0/0),(0/1) and (1/1) and show that, in our data, they are in fact not different from each other (Supplementary Figure 8). We thus conclude that the MMR efficiency in carriers is decreased relative to non-carriers, and therefore their mutational load should be increased.

Supplementary note 10: Effects of microsatellite mutator alleles on sDNMs

Somatic and germline mutations in mismatch-repair genes are known to cause microsatellite instability and hypermutator phenotypes in tumors of the colon and endometrium. Sequencing of large cohorts of tumors has revealed several mutational signatures associated with mismatch repair deficiency[32].

These signatures show a strong correlation with somatic microsatellite instability. Mutations in components of the base-excision repair pathway, most notably *NTHL1* and *MUTYH,* also cause distinct mutational signatures in tumors with these mutations (COSMIC signatures SBS30 and SBS36[32], respectively). Given the effects of rs4987188[A] and rs8191642[G] on the rate of mDNMs, we were interested in knowing if they also affect the mutational spectra of single-base-substitution DNMs, for example by altering the MMR/BER pathways in the testis.

Using only phased DNMs, we compared the 96-class trinucleotide spectra of DNMs transmitted from carrier and non-carrier mothers and fathers. All spectra were highly similar (pairwise cosine similarities >0.95) and showed no hint of MMR deficient related mutational signatures (Supplementary Figure 9). This suggests that the effects of rs4987188[A] and rs8191642[G] are confined to microsatellite sites and they do not otherwise affect the fidelity of the mismatch or base-excision repair pathways.

Supplementary Figures

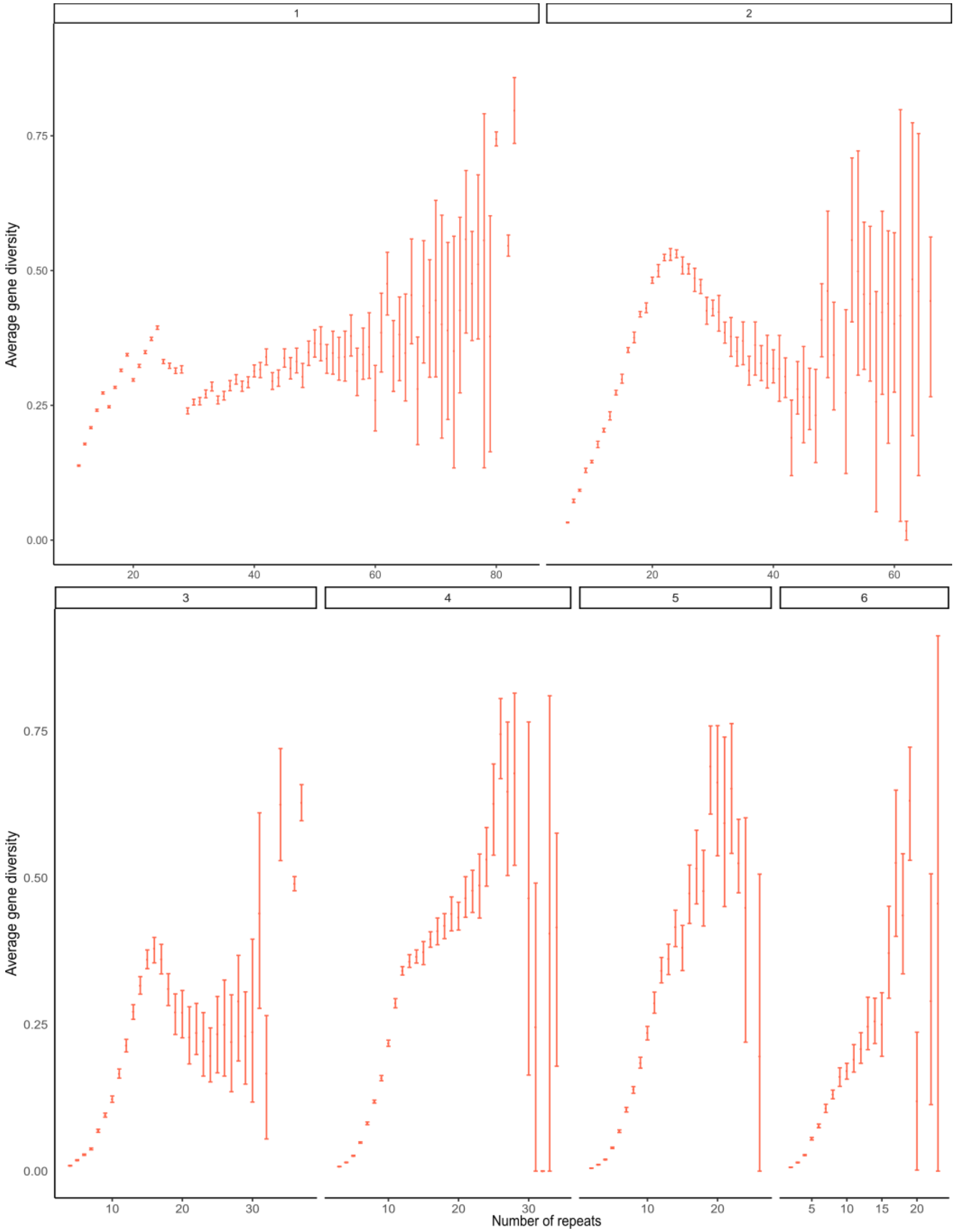

Supplementary Figure 1 | **Expected heterozygosity vs number of repeats in UKB.** Average expected heterozygosity in UKB as a function of repeat number stratified on motif length with

error bars representing 95% confidence intervals. The drop in all motif lengths is most likely due our inability to reliably detect long alleles from short reads, causing underestimation of expected heterozygosity values at microsatellite with long reference alleles (n1bp=753,664; n2bp=330,797; n3bp=203,673; n4bp=442,153; n5bp=322,598; n6bp=338,919).

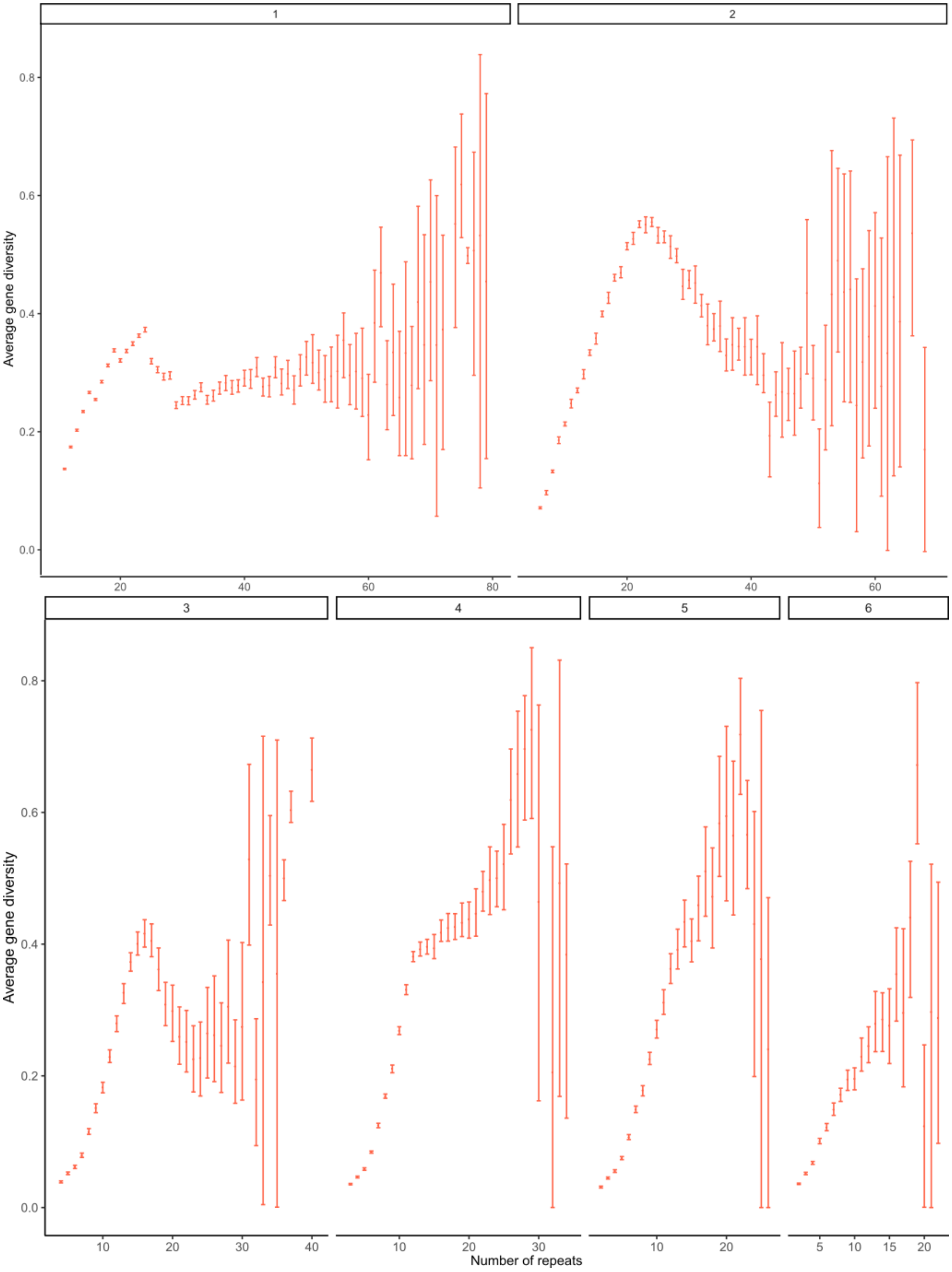

Supplementary Figure 2 | **Expected heterozygosity vs number of repeats in Iceland.** Average expected heterozygosity in Icelandic data set as a function of repeat number stratified on motif length with error bars representing 95% confidence intervals (n1bp=705,712; n2bp=232,770; n3bp=81,157; n4bp=183,244; n5bp=99,169; n6bp=89,878).

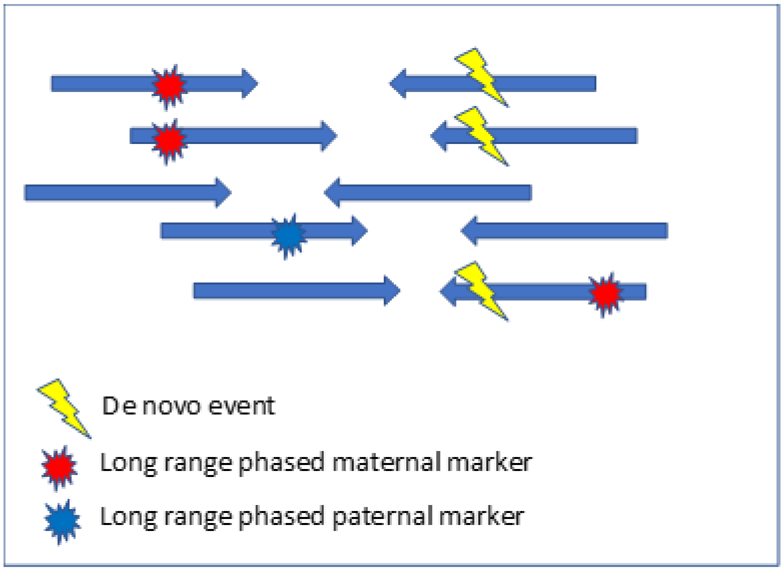

Supplementary Figure 3 | **Read pair mDNM phasing.** We use read pairs which contain a long range phased marker and report an mDNM. Reads with long range phased markers covering the de novo site but supporting the other allele can also further give information on the parent of origin. An example of a maternal mDNM where three read pairs report a long range phased maternal marker and a de novo allele, one pair is not informative and one contains a long range phased paternal marker and not the de novo allele.

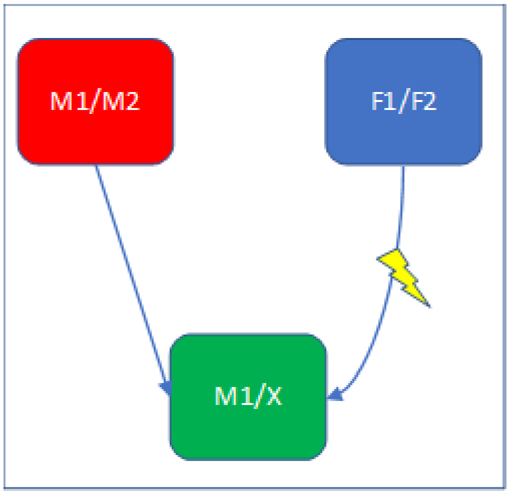

Supplementary Figure 4 | **Allele based mDNM phasing**. If the de novo allele is present in neither parent and the other allele is present only in one parent we phase the de novo event to the other parent. Here we phase the de novo event to the father since a maternal allele is seen in the offspring but neither of the paternal ones.

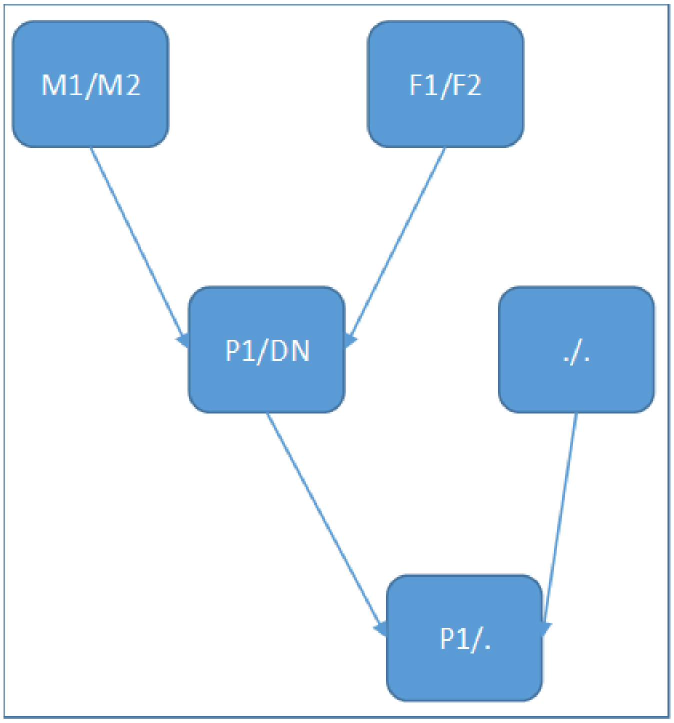

Supplementary Figure 5 | **mDNM phasing using Haplotype sharing in three generation families**. If the de novo is transmitted from the offspring to its child we phase the de novo to the parent sharing a haplotype with the offspring, and to the parent not sharing a haplotype if de novo is not transmitted.

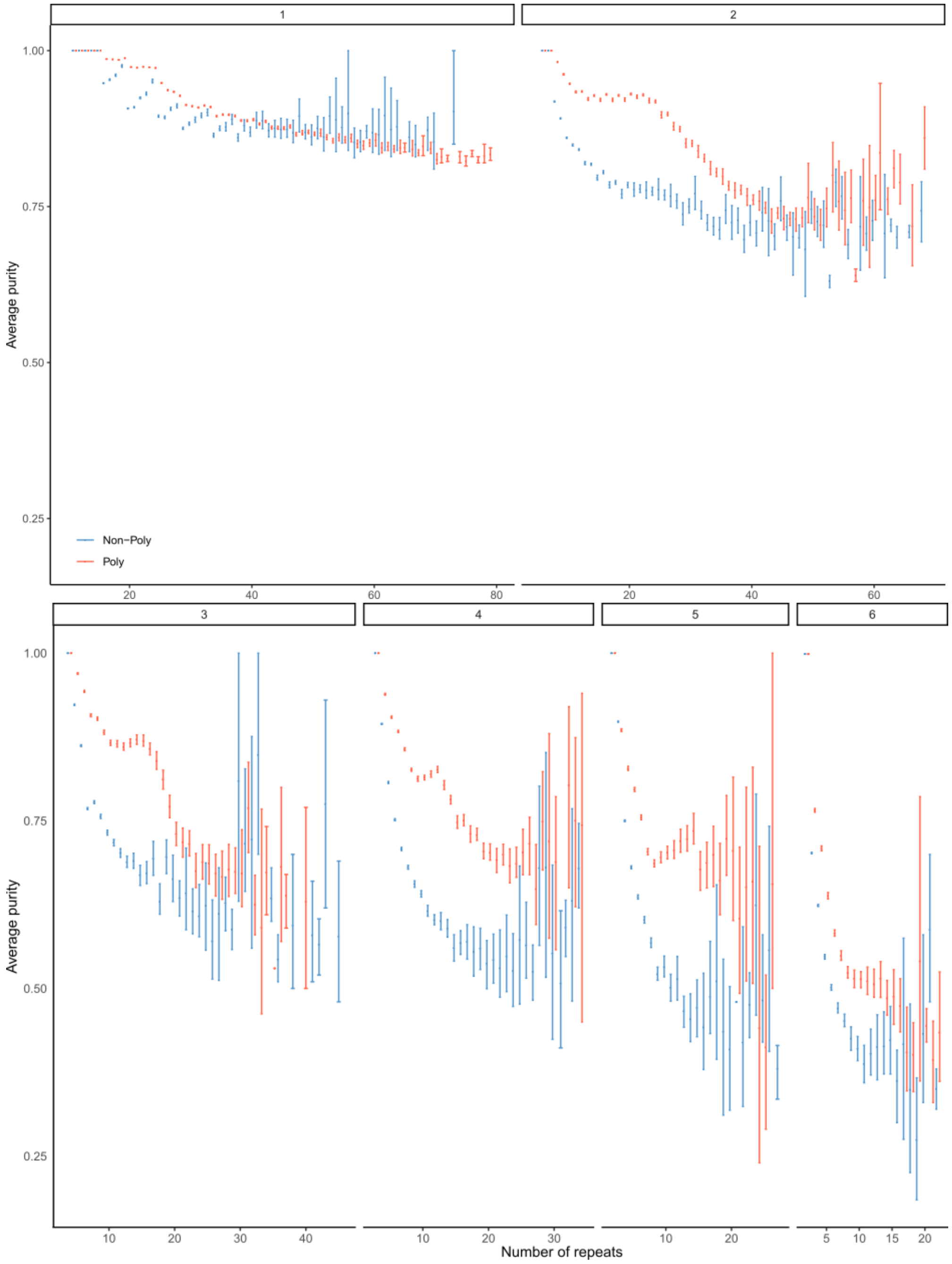

Supplementary Figure 6 | **Average repeat purity vs repeat number in Iceland.** Average repeat purity as a function of repeat number stratified on motif length for the Icelandic data set with error bars representing 95% confidence intervals. Red marks polymorphic STRs (microsatellites) and blue non-polymorphic STRs (n1bp=poly:705,712/non-poly:33,678; n2bp=poly:232,770/non-poly:136,900; n3bp=poly:81,157/non-poly:237,062; n4bp=poly:183,244/non-poly:626,544; n5bp=poly:99,169/non-poly:1,296,377; n6bp=poly:89,878/non-poly:1,536,977).

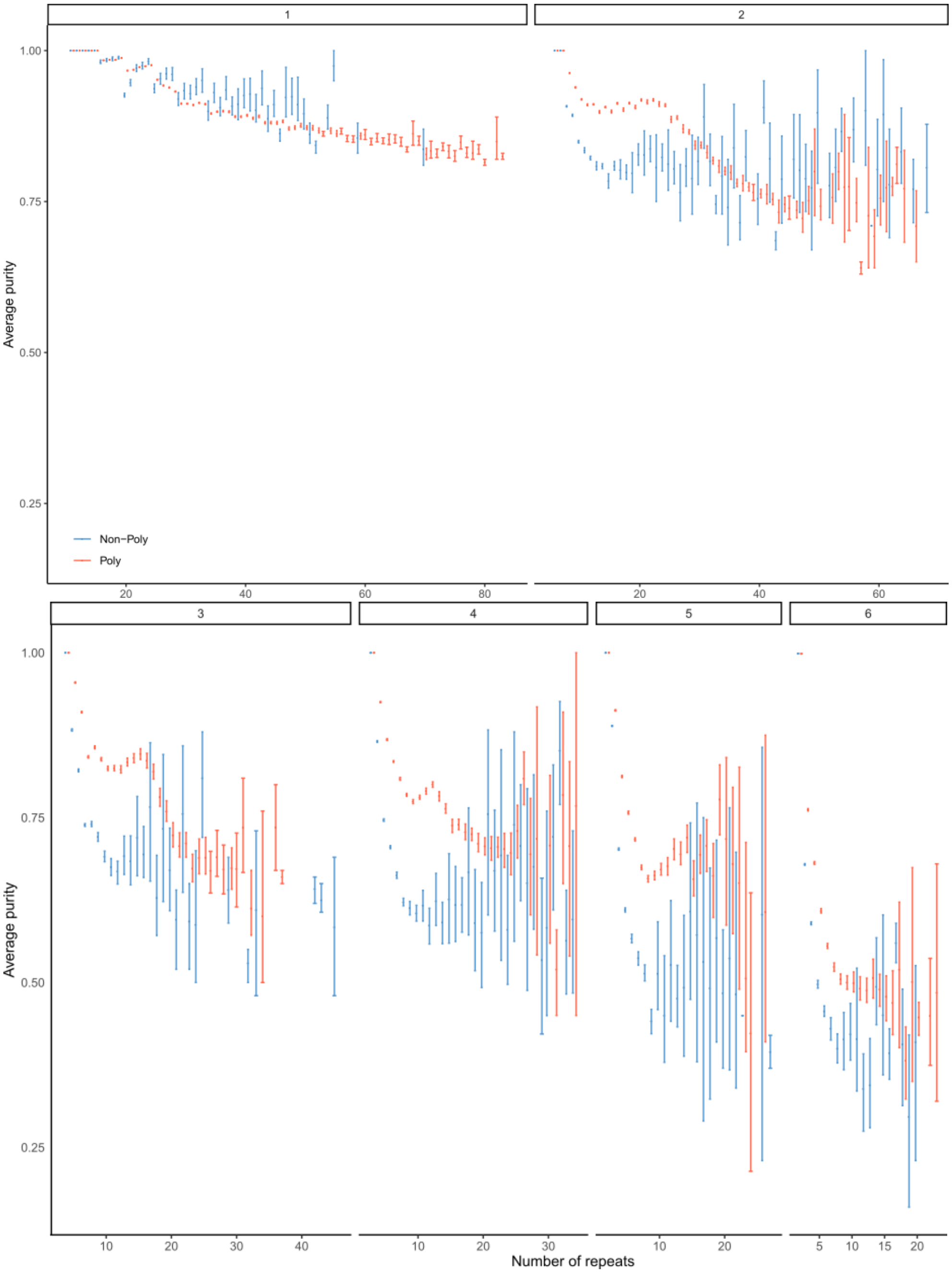

Supplementary Figure 7 | **Average repeat purity vs repeat number in UKB.** Average repeat purity as a function of repeat number stratified on motif length for the UKB data set with error bars representing 95% confidence intervals. Red marks polymorphic STRs (microsatellites) and blue non-polymorphic STRs (n1bp=poly:753,664/non-poly:5,951; n2bp=poly:330,797/non-poly:34,362; n3bp=poly:203,673/non-poly:113,662; n4bp=poly:442,153/non-poly:363,128; n5bp=poly:322,598/non-poly:1,067,405; n6bp= poly:338,919/non-poly:1,284,871).

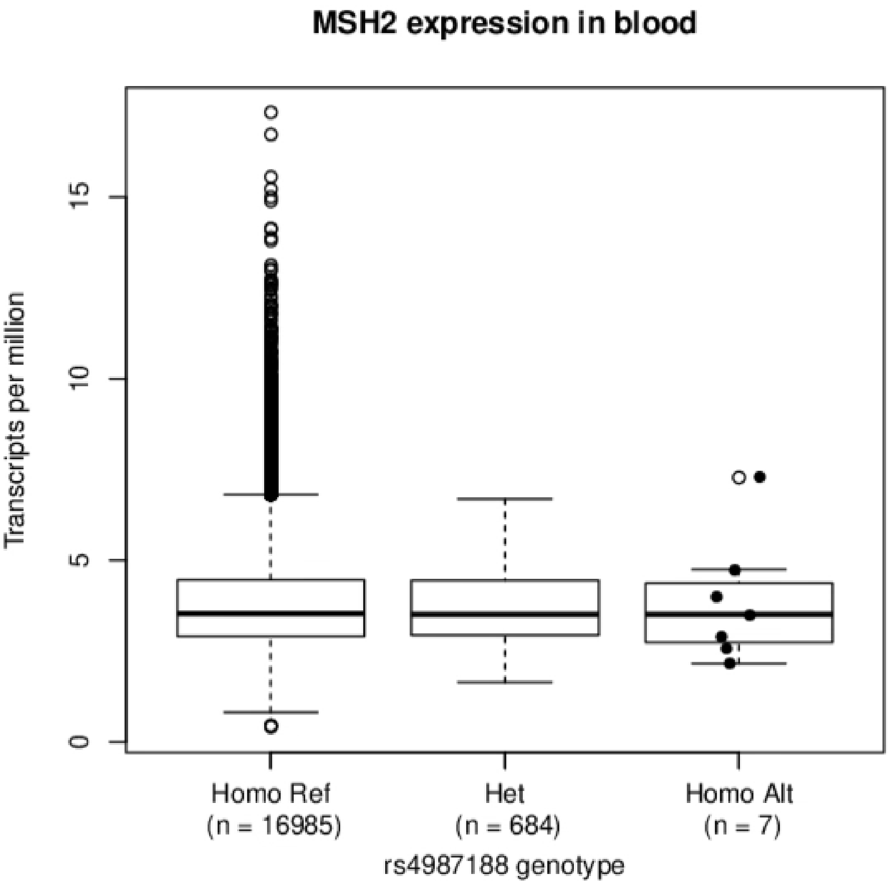

Supplementary Figure 8 | **Boxplot for MSH2 RNA-expression of all rs4987188 genotypes.** They do not have significant differences and strengthen our assumptions that carriers should have decreased MMR-efficiency relative to non-carriers and thus transmit more mDNMs to their offspring. White boxes indicate quantile boundary with the black horizontal line showing the median and edges representing the 25th and 75th percentiles. Whiskers extend to the smallest/largest value that is no less/more than 1.5 times the interquartile range from the median. The filled circles correspond to expression values, for outliers that lie beyond the extremes of the whiskers.

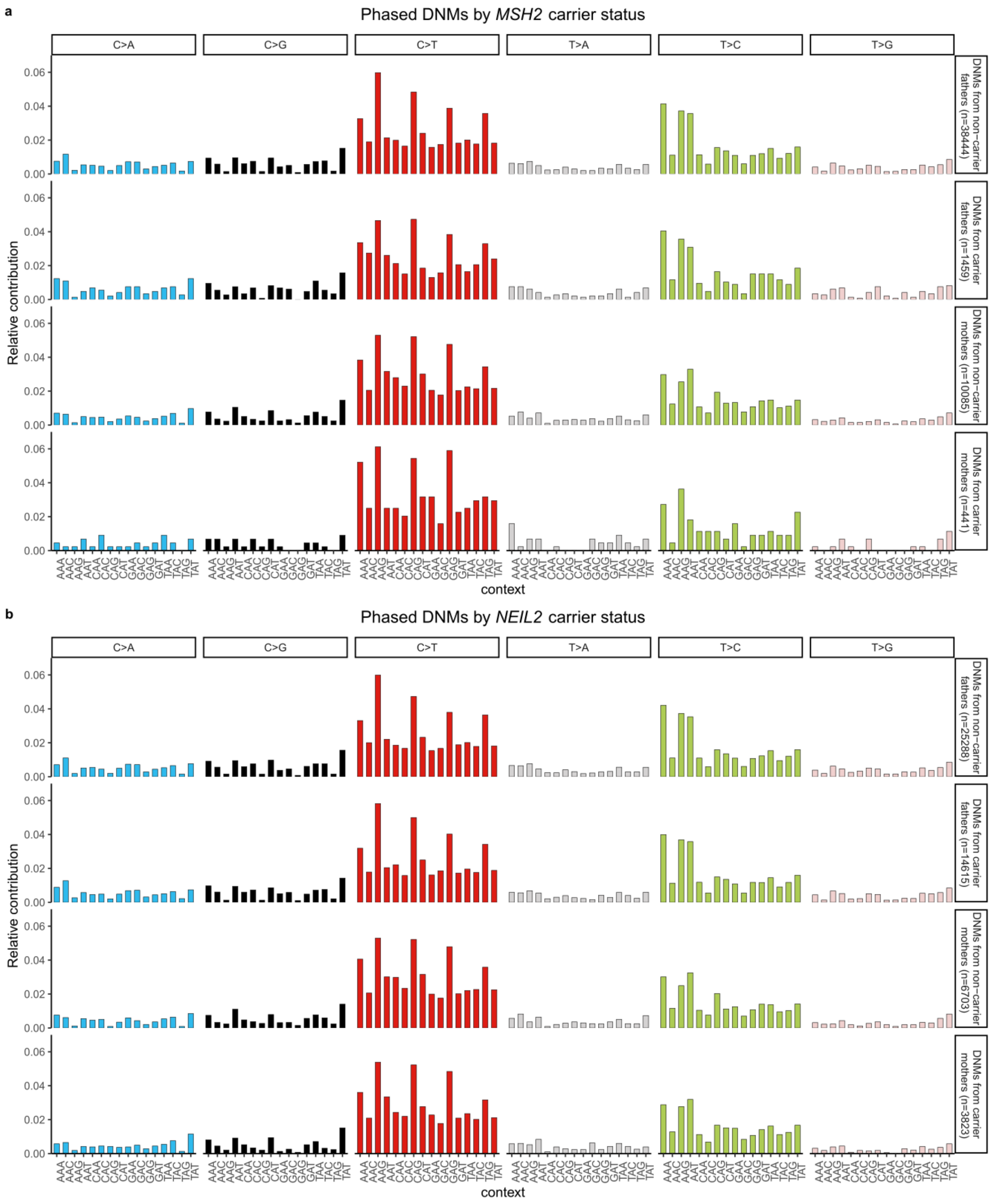

Supplementary Figure 9 | **sDNM mutation signatures.** sDNM mutation signatures for offspring of paternal and maternal carriers and non-carriers of both rs4987188 (panel a) and rs8191642 (panel b). The signatures are not different in any of the four comparisons so we assume that the variants do not affect the generation of sDNMs.

Supplementary Tables

Class representative	Other members
A	T
C	G
AC	CA, GT, TG
AG	GA, CT, TC
AT	TA
CG	GC
AAC	ACA, CAA, GTT, TGT, TTG
AAG	AGA, GAA, CTT, TCT, TTC
AAT	ATA, TAA, ATT, TAT, TTA
ACC	CAC, CCA, GGT, GTG, TGG
ACG	CGA, GAC, CGT, TCG, GTC
ACT	CTA, TAC, AGT, TAG, GTA
AGC	GCA, CAG, GCT, TGC, CTG
AGG	GAG, GGA, CCT, CTC, TCC
ATC	TCA, CAT, GAT, TGA, ATG
CCG	CGC, GCC, CGG, GCG, GGC

Supplementary Table 1 | **Motif equivalence classes**. Representative motif for each motif equivalence class and its members.

RRT length	Purity effect (Ice/UKB)	*P* (Ice/UKB)
11-20	0.39/0.23	$< 1.0 \cdot 10^{-320}$/$< 1.0 \cdot 10^{-320}$
21-30	0.71/0.67	$< 1.0 \cdot 10^{-320}$/$< 1.0 \cdot 10^{-320}$
31-40	0.87/0.85	$< 1.0 \cdot 10^{-320}$/$< 1.0 \cdot 10^{-320}$
41-50	1.17/1.15	$< 1.0 \cdot 10^{-320}$/$< 1.0 \cdot 10^{-320}$
51-60	1.17/1.18	$< 1.0 \cdot 10^{-320}$/$< 1.0 \cdot 10^{-320}$
61-70	1.10/1.13	$< 1.0 \cdot 10^{-320}$/$< 1.0 \cdot 10^{-320}$
71-80	1.05/1.08	$< 1.0 \cdot 10^{-320}$/$< 1.0 \cdot 10^{-320}$
81-90	0.96/1.07	$3.9 \cdot 10^{-141}$/$6.8 \cdot 10^{-183}$
>90	0.76/0.83	$5.4 \cdot 10^{-56}$/$6.1 \cdot 10^{-57}$

Supplementary Table 2 | **Repeat purity regression effects on expected heterozygosity.** Linear regression coefficients and p-values for the effect of repeat purity on expected heterozygosity within ten base pair bins of RRT length for both the Icelandic and UKB datasets. P-values and coefficients were extracted from R logistic regression models generated with the glm function.

RRT length	Purity effect (Ice/UKB)	*P* (Ice/UKB)
11-20	7.86/5.31	$< 1.0 \cdot 10^{-320}$/$< 1.0 \cdot 10^{-320}$
21-30	7.84/6.09	$< 1.0 \cdot 10^{-320}$/$< 1.0 \cdot 10^{-320}$
31-40	6.11/5.47	$< 1.0 \cdot 10^{-320}$/$< 1.0 \cdot 10^{-320}$
41-50	5.94/4.96	$< 1.0 \cdot 10^{-320}$/$< 1.0 \cdot 10^{-320}$
51-60	4.78/3.668	$< 1.0 \cdot 10^{-320}$/$2.4 \cdot 10^{-83}$
61-70	4.42/2.72	$2.8 \cdot 10^{-164}$/$6.0 \cdot 10^{-17}$
71-80	4.22/1.95	$2.0 \cdot 10^{-61}$/$7.1 \cdot 10^{-6}$
81-90	4.03/-0.09	$6.2 \cdot 10^{-26}$/0.9
>90	2.72/0.61	$1.0 \cdot 10^{-16}$/0.1

Supplementary Table 3 | **Repeat purity regression effects on polymorphism rate.** Logistic regression coefficients and p-values for the effect of repeat purity on polymorphism rate within ten base pair bins of RRT length for both the Icelandic and UKB datasets. P-values and coefficients were extracted from R logistic regression models generated with the glm function.

Motif length	Effect (Ice/UKB)	*P* (Ice/UKB)
1	0.15/0.17	$4.5 \cdot 10^{-312}$/< $1.0 \cdot 10^{-320}$
2	0.14/0.10	< $1.0 \cdot 10^{-320}$/< $1.0 \cdot 10^{-320}$
3	-0.12/-0.05	< $1.0 \cdot 10^{-320}$/< $1.0 \cdot 10^{-320}$
4	-0.07/-0.04	$1.1 \cdot 10^{-155}$/< $1.0 \cdot 10^{-320}$
5	-0.06/-0.03	$8.0 \cdot 10^{-141}$/< $1.0 \cdot 10^{-320}$
6	-0.02/-0.01	$3.9 \cdot 10^{-18}$/$1.52 \cdot 10^{-95}$

Supplementary Table 4 | **GC content regression effects on expected heterozygosity.** Linear regression coefficients and p-values for the effect of GC motif content on expected heterozygosity within motif lengths for both the Icelandic and UKB datasets. P-values and coefficients were extracted from R logistic regression models generated with the glm function.

Motif length	Effect (Ice/UKB)	*P* (Ice/UKB)
1	-0.20/-1.33	$4.9 \cdot 10^{-3}/4.6 \cdot 10^{-41}$
2	-0.29/-0.21	$1.3 \cdot 10^{-79}/1.6 \cdot 10^{-15}$
3	-0.79/-1.12	$< 1.0 \cdot 10^{-320}/< 1.0 \cdot 10^{-320}$
4	-1.47/-1.97	$< 1.0 \cdot 10^{-320}/< 1.0 \cdot 10^{-320}$
5	-2.02/-1.67	$< 1.0 \cdot 10^{-320}/< 1.0 \cdot 10^{-320}$
6	-1.55/-1.34	$< 1.0 \cdot 10^{-320}/< 1.0 \cdot 10^{-320}$

Supplementary Table 5 | **GC content regression effects on polymorphism rate.** Logistic regression coefficients and p-values for the effect of GC motif content on polymorphism rate within motif lengths for both the Icelandic and UKB datasets. P-values and coefficients were extracted from R logistic regression models R generated with the glm function.

Motif length (bp)	Minimum RRT (bp)
1	10
2	8
3	9
4	8
5	10
6	12

Supplementary Table 6 | **Repeat length requirements.** Minimum reference repeat tract length per motif length.

Chrom	Pos	Motif	Ref. (No repeats)	Offspring gt	Father gt	Mother gt	Verified
chr11	80254522	CTAT	15	15/15	11/16	15/15	yes
chr12	100919737	TATC	15	15/18	15/17	15/17	yes
chr2	199703012	GTTT	8	9/9	8/10	8/9	yes
chr4	115864579	TG	11	11/13	11/12	11/11	no
chr7	86946160	CA	16	14/18	16/18	15/18	yes
chr11	43435069	AGAT	16	17/17	15/18	15/17	yes
chr13	54157465	TA	20	16/19	18/21	16/22	yes
chr14	82746964	AATA	8	8/10	8/9	8/9	yes
chr17	54067449	TTTTA	7	7/8	7/7	7/7	yes
chr18	3742950	CTTC	11	11/14	11/12	11/13	yes
chr18	63468251	AAC	8	10/12	8/10	10/10	yes
chr1	10498281	AAAAC	8	8/7	9/9	7/9	yes
chr4	140179641	TGTTT	8	7/9	8/5	5/7	yes
chr6	44850746	ATCT	13	16/16	15/15	10/16	yes
chr16	69147179	AAT	15	14/16	15/15	15/14	yes
chr1	180853627	AC	21	19/25	19/26	19/20	yes
chr20	34877898	AGAT	12	15/16	13/16	16/16	yes
chr3	85784531	TATC	13	13/12	10/14	11/12	yes
chr4	169443052	TATC	13	12/15	14/14	13/12	yes
chr6	38464486	TTTTC	6	6/5	6/7	6/7	yes
chr7	29184144	AGAT	14	14/15	11/16	14/13	yes
chr8	110980554	ATCT	10	12/12	11/11	12/13	yes
chr11	22272303	CA	22	22/19	23/26	22/25	yes
chr12	29413662	AC	15	16/20	14/16	14/19	yes
chr13	80326450	TG	9	9/8	8/8	8/8	yes
chr1	172791800	AT	7	7/14	7/7	7/13	yes
chr8	19119757	TA	9	9/13	9/12	9/12	yes

Supplementary Table 7 | **mDNM pacBio verification.** mDNMs verified by haplotype resolved assemblies generated from pacBio HiFi sequencing data. We were unable to verify homopolymer mDNMs due to high error rates.

Motif length (bp)	Shared	Not shared	Error rate
1	47	6	11.3%
2	108	6	5.3%
3	16	1	5.9%
4	44	0	0%
5	2	0	0%
6	0	0	0%

Supplementary Table 8 | **mDNM sharing between monozygotic twins.** Sharing of mDNMs between monozygotic twins stratified on motif length.

Motif length (bp)	Transmission rate	Error estimate
1	0.40 (861/2,156)	20%
2	0.50 (2,558/5,083)	0%
3	0.51 (433/857)	2%
4	0.53 (1,458/2,764)	6%
5	0.55 (169/310)	10%
6	0.47 (27/58)	6%
Total	0.49 (5,506/11,228)	2%

Supplementary Table 9 | **Transmission rate mDNM verification.** Transmission rate by motif length and combined for all mDNMs phased using haplotype sharing in three-generation families.

		Read pair tracing		
		Paternal	Maternal	Total
3 generation	Paternal	2,199	70	2,269(75.5%)
	Maternal	118	619	737(24.5%)
	Total	2,317(77.1%)	689(22.9%)	

Supplementary Table 10 | **mDNM phasing concordance between three generation and read pair phasing.** Comparison of phasing results for mDNMs phased using both three generation and read pair phasing. The concordance between the methods is 93.7% and the ratio between maternal and paternal mDNMs is similar in both sets.

		Allele based		
		Paternal	Maternal	Total
3 generation	Paternal	2,390	39	2,429(78.2%)
	Maternal	103	576	679(21.8%)
	Total	2,493(80.2%)	615(19.8%)	

Supplementary Table 11 | **mDNM phasing concordance between three generation and allele based phasing.** Comparison of phasing results for mDNMs phased using both three generation and allele based phasing. The concordance between the methods is 95.4% and the ratio between maternal and paternal mDNMs is similar in both sets.

		Read pair tracing		
		Paternal	Maternal	Total
Allele based	Paternal	8,865	9	8,874(79.6%)
	Maternal	21	2,258	2,279(20.4%)
	Total	8,886 (79.7%)	2,267(20.3%)	

Supplementary Table 12 | **mDNM phasing concordance between read pair and allele based phasing.** Comparison of phasing results for mDNMs phased using both read pair and allele based phasing. The concordance between the methods is 99.7%.

Motif length (bp)	Maternal	Paternal	Maternal percentage
1	2,948	5,141	36.4%
2	4,084	17,388	19.0%
3	883	2,663	24.9%
4	2,354	9,184	20.4%
5	341	977	25.9%
6	60	148	28.8%
Total	10,670	35,501	23.1%

Supplementary Table 13 | **Parental ratios of mDNMs.** Parental ratios of mDNMs stratified on motif length and on the full set.

Motif length (bp)	Odds ratio maternal/paternal (95% CI)	Two-sided Fisher exact test *P*
1	2.25 (2.14-2.37)	$2.0 \cdot 10^{-198}$
2	0.65 (0.62-0.68)	$6.3 \cdot 10^{-84}$
3	1.11 (1.03-1.20)	$1.0 \cdot 10^{-2}$
4	0.81 (0.77-0.85)	$4.2 \cdot 10^{-16}$
5	1.17 (1.03-1.32)	$1.7 \cdot 10^{-2}$
6	1.39 (1.03-1.86)	$3.0 \cdot 10^{-2}$
Motif length (without 1bp)	Odds ratio maternal/paternal (95% CI) (no 1 bp)	Two-sided Fisher exact test *P* (no 1 bp)
2	0.84 (0.80-0.88)	$1.0 \cdot 10^{-11}$
3	1.34 (1.24-1.45)	$3.3 \cdot 10^{-12}$
4	1.01 (0.95-1.06)	0.78
5	1.39 (1.22-1.57)	$6.3 \cdot 10^{-7}$
6	1.64 (1.21-2.20)	$1.9 \cdot 10^{-3}$

Supplementary Table 14 | **Parental mDNM ratios.** Per motif length enrichment of mDNMs between maternally and paternally phased mDNMs. Top half shows enrichment including homopolymers and bottom half without them. Tri-, penta- and hexanucleotide repeats are enriched in maternal mDNMs while dinucleotide microsatellites are paternally enriched.

Mutation size (In motifs)						
Motif	<-2 (p/m)	-2 (p/m)	-1 (p/m)	1 (p/m)	2 (p/m)	>2 (p/m)
1	312(168/144)	140(69/71)	2,782(1,671/1,111)	3,962(2,777/1,185)	214(121/93)	679(335/344)
2	610(399/211)	2,482(2,101/381)	7,124(6,192/932)	8,933(6,936/1,997)	1,849(1,490/359)	474(270/204)
3	90(52/38)	160(117/43)	1,516(1,053/463)	1,524(1,251/273)	228(171/57)	28(19/9)
4	134(90/44)	237(170/67)	5,229(3,972/1,257)	5,798(4,861/937)	107(73/34)	33(18/15)
5	14(11/3)	44(30/14)	583(392/191)	651(533/118)	13(7/6)	13(4/9)
6	0(0/0)	4(3/1)	79(50/29)	117(90/27)	6(3/3)	2(2/0)
Total	1,160(720/440)	3,067(2,490/577)	17,313(13,330/3,983)	20,985(16,448/4,573)	2,417(1,865/552)	1,229(648/576)

Supplementary Table 15 | **Parental comparison of mDNM sizes.** Counts of mutation sizes of phased mDNMs per motif length and for all motif lengths combined. Maternal and paternal counts are given in brackets. Maternal mDNMs are larger on average than paternal ones and we see the ratio of maternal mDNMs increases with their size.

Motif length (bp)	Mean RRT length(bp) (pat/mat)	*P*
1	17.0/16.9	0.12
2	39.2/37.2	**$1.9 \cdot 10^{-24}$**
3	40.8/40.4	0.72
4	52.2/50.1	**$5.6 \cdot 10^{-9}$**
5	54.7/51.1	**$1.6 \cdot 10^{-3}$**
6	49.8/42.6	**$2.2 \cdot 10^{-2}$**

Supplementary Table 16 | **Parental comparison of RRT length at mDNMs.** Mean RRT lengths for paternal and maternal mDNMs. The paternal mDNMs have longer RRT at di-, tetra-, penta- and hexanucleotide microsatellites (Mann-Whitney U-test). Bold represents significant difference in RRT length between paternal and maternal mDNMs ($p<0.05$).

Motif equivalence class	% of paternal	% of maternal	Odds ratio maternal/paternal (*P*) - test
AAC	10.7%	7.6%	**0.68[0.52;0.90] ($7.6 \cdot 10^{-3}$) – X^2**
AAG	2.1%	2.7%	1.28[0.77;2.05] (0.36) – fisher
AAT	80.9%	84.2%	**1.26[1.03;1.55] (0.02) – X^2**
ACC	0.3%	0.0%	No maternal mDNMs.
ACG	0.0%	0.0%	No mDNMs
ACT	1.6%	0.8%	0.49[0.20;1.04] ($7.2 \cdot 10^{-2}$) – X^2
AGC	0.6%	1.4%	2.14[0.99;4.50] ($5.1 \cdot 10^{-2}$) – X^2
AGG	0.2%	0.2%	1.56[0.19;8.48] (0.64) – fisher
ATC	3.4%	2.1%	0.63[0.37;1.02] ($7.2 \cdot 10^{-2}$) – X^2
CCG	0.3%	1.0%	**3.39[1.28;9.20] ($2.0 \cdot 10^{-2}$) – fisher**
Motif equivalence class	% of paternal	% of maternal	Enrichment
AC	88.7%	79.8%	**0.50[0.46;0.55] ($2.9 \cdot 10^{-48}$) – fisher**
AG	4.6%	7.4%	**1.65[1.43;1.88] ($4.5 \cdot 10^{-12}$) – fisher**
AT	6.6%	12.7%	**2.05[1.84;2.29] ($3.6 \cdot 10^{-35}$) – fisher**
CG	0.04%	0.1%	3.04[0.88;9.76] ($6.2 \cdot 10^{-2}$) – fisher

Supplementary Table 17 | **Parental comparison of motif equivalence classes.** Motif equivalence class odds ratios for di- and trinucleotide microsatellites. Maternal mDNMs are more common at AAT, CCG, AG and AT motif class microsatellites while paternal mDNMs are more common at AAC and AC motif class microsatellites. Bold represents significant enrichment ($p<0.05$) from the statistical test applied (two-sided Fisher or X^2 depending on the number of mDNMs from each class).

Members of ATTCT motif equivalence class
ATTCT
TTCTA
TCTAT
CTATT
TATTC
AGAAT
GAATA
AATAG
ATAGA
TAGAA

Supplementary Table 18 | **ATTCT motif equivalence class.** Members of the ATTCT motif equivalence class considered for paternal expansion bias.

Marker 1	Marker 2	Conditional *P* 1-2	Conditional *P* 2-1	R^2
**chr2:47416318**	chr2:47494068	$8.9 \cdot 10^{-2}$	$5.1 \cdot 10^{-2}$	0.74
**chr2:47416318**	chr2:47491330:0	$3.6 \cdot 10^{-2}$	$2.3 \cdot 10^{-2}$	0.60
**chr2:47416318**	chr2:47491330:1	$3.6 \cdot 10^{-2}$	$2.3 \cdot 10^{-2}$	0.60
chr2:47494068	chr2:47491330:0	0.17	0.18	0.80
chr2:47494068	chr2:47491330:1	0.17	0.18	0.80
chr2:47491330:0	chr2:47491330:1	1.00	1.00	1.00

Supplementary Table 19 | **Conditional association for *MSH2* signal.** Conditional association p-values for markers significantly associating with increased mDNM rate (*MSH2* missense marker in bold). All markers are correlated but a residual signal remains after conditioning the intergenic signal at chr2:47491330 for the missense marker.

Population	Allele frequency of rs4987188
Iceland	1.9%
UKB	1.5%
Finngen	3.0%
Africa	0.2%
Asia	0.0.%

Supplementary Table 20 | **Allele frequencies of rs4987188 in different populations.** It is most frequent in the Finnish population and not found in Asians.

	R^2 (*P*)
Paternally transmitted	0.03 (0.32)
Maternally transmitted	-0.04 (0.2)

Supplementary Table 21 | **mDNM and sDNM correlation.** Correlation between paternally transmitted sDNMs and mDNMs and between maternally transmitted sDNMs and mDNMs.

Motif	Repeat tract length	Purity	GC content	Motif length
All	0.10 ($<1 \cdot 10^{-320}$)	0.12($<1 \cdot 10^{-320}$)	$1.3 \cdot 10^{-2}$(0.46)	-0.15 ($<1 \cdot 10^{-320}$)
1bp	0.16($<1 \cdot 10^{-320}$)	0.28($<1 \cdot 10^{-320}$)	0.02($<1 \cdot 10^{-320}$)	X
2 bp	0.10($<1 \cdot 10^{-320}$)	0.17($<1 \cdot 10^{-320}$)	-0.01($4.9 \cdot 10^{-87}$)	X
3 bp	0.12 ($<1 \cdot 10^{-320}$)	0.14 ($<1 \cdot 10^{-320}$)	-0.03($3.6 \cdot 10^{-235}$)	X
4 bp	0.10($<1 \cdot 10^{-320}$)	0.10 ($<1 \cdot 10^{-320}$)	-0.003($9.8 \cdot 10^{-11}$)	X
5 bp	0.10 ($<1 \cdot 10^{-320}$)	0.05($1.1 \cdot 10^{-121}$)	-0.003($2.3 \cdot 10^{-2}$)	X
6 bp	0.09($<1 \cdot 10^{-320}$)	0.02($2.0 \cdot 10^{-18}$)	$4.1 \cdot 10^{-4}$ (0.87)	X

Supplementary Table 22 | **mDNM rate regression effects of microsatellite attributes.** Poisson multiple regression coefficients and p-values for the effect on the mDNM rate by RRT length, repeat purity, GC content and motif length for all markers and stratified on motif length. Repeat tract length and repeat purity remain significant and consistent in their effect directionality across the full data set and all motif length subsets. GC-content is positively correlated to the mDNM rate for homopolymers but for di-, tri-, tetra-, and pentanucleotide repeats the motif GC-content has an inverse correlation to the mDNM rate.

Phasing method	Paternal effect	*P*	Maternal effect	*P*
Allele based	0.17 [0.15;0.18]	$1.2 \cdot 10^{-102}$	0.07 [0.05;0.08]	$5.1 \cdot 10^{-16}$
Read back	0.16 [0.15;0.18]	$3.6 \cdot 10^{-101}$	0.07 [0.06;0.09]	$7.1 \cdot 10^{-21}$
3 generation	0.18 [0.15;0.21]	$9.8 \cdot 10^{-34}$	0.05 [0.03;0.07]	$1.7 \cdot 10^{-6}$

Supplementary Table 23 | **mDNM age effects per phasing method.** Poisson regression coefficients and p-values for subsets of phased markers by each method applied. The effects for allele based phasing and read back phasing match but the three generation phasing subset gives a stronger paternal effect and a weaker maternal one. P-values computed using one sided X^2 test.

Motif class	Maternal	Paternal	Full marker set	TRF output
A	97.5%	97.7%	99.2%	99.2%
C	2.5%	2.3%	0.8%	0.8%
AC	67.6%	84.3%	49.1%	54.4%
AG	8.9%	5.6%	19.9%	23.4%
AT	23.2%	10.0%	30.5%	21.7%
CG	0.4%	0.1%	0.4%	0.4%
AAC	13.0%	13.3%	27.1%	20.8%
AAG	3.8%	2.0%	15.0%	10.0%
AAT	75.3%	77.6%	34.9%	32.9%
ACC	0.8%	0.4%	4.0%	7.4%
ACG	0.0%	0.0%	0.1%	0.1%
ACT	1.2%	0.9%	0.9%	1.9%
AGC	2.1%	1.2%	3.0%	7.8%
AGG	0.8%	0.6%	7.5%	11.3%
ATC	2.4%	3.9%	4.3%	6.1%
CCG	0.6%	0.2%	3.1%	1.6%

Supplementary Table 24 | **Motif equivalence class composition.** Fraction of each motif equivalence class within homopolymers, di- and trinucleotide repeats split on maternal and paternal mDNMs, fraction of each motif equivalence class in full marker set and in the Tandem Repeats Finder (TRF) output.

Detection frequency	DNM rate estimate
$9.0 \cdot 10^{-6}$	$5.0 \cdot 10^{-5}$
$7.6 \cdot 10^{-5}$	$5.6 \cdot 10^{-5}$
$1.0 \cdot 10^{-2}$	$8.1 \cdot 10^{-5}$
$5.0 \cdot 10^{-2}$	$9.3 \cdot 10^{-5}$
0.1	$1.0 \cdot 10^{-4}$

Supplementary Table 25 | **mDNM rates per detection frequency.** Microsatellite mDNM rate estimates for different detection frequencies, the number of markers included in the analysis depends on the detection frequency defined by the sample size.

Motif equivalence class	Regression α and (*P*) for comparison with AC-class mDNM rate
AG	-1.20 ($<1 \cdot 10^{-320}$)
AT	-1.08 ($<1 \cdot 10^{-320}$)
CG	-0.43 ($7.7 \cdot 10^{-3}$)

Supplementary Table 26 | **Regression effects of motif equivalence class on mDNM rate.** Regression coefficients and p-values from a Poisson regression comparing the mDNM rate of the other 2bp motif equivalence classes to the AC class using available trios per marker as exposure but without correcting for RRT length. All motif classes have significantly lower mDNM rates. P-values and coefficients were extracted from R Poisson regression models generated with the glm function.

Motif equivalence class	Regression α and (*P*) for comparison with CG-class mDNM rate
AC	-0.81 ($5.7 \cdot 10^{-7}$)
AG	-1.48 ($1.7 \cdot 10^{-19}$)
AT	-1.18 ($5.8 \cdot 10^{-13}$)

Supplementary Table 27 | **Regression effects of motif equivalence class on mDNM rate corrected for RRT length.** Regression coefficients and p-values from a Poisson regression comparing the mDNM rate of the other motif equivalence classes to the CG class after correcting for RRT length and using available trios per marker as exposure. All motif classes have significantly lower mDNM rates. P-values and coefficients were extracted from R Poisson regression models generated with the glm function.

Motif equivalence class	Regression α (*P*) for comparison with AAT mDNM rate
AAC	-1.35 ($2.7 \cdot 10^{-217}$)
AAG	-1.67 ($5.3 \cdot 10^{-75}$)
ACC	-2.99 ($5.6 \cdot 10^{-29}$)
ACG	-0.06 (0.92)
ACT	-2.49 ($3.3 \cdot 10^{-88}$)
AGC	-1.8 ($8.6 \cdot 10^{-41}$)
AGG	-3.09($4.9 \cdot 10^{-37}$)
ATC	-1.78 ($1.2 \cdot 10^{-120}$)
CCG	-1.14($1.7 \cdot 10^{-11}$)

Supplementary Table 28 | **Regression effects of motif equivalence class on mDNM rate.** Regression coefficients and p-values from a Poisson regression comparing the mDNM rate of the other 3bp motif equivalence classes to the AAT class, correcting for RRT length and using available trios per marker as exposure. All motif classes expect for the rarest one (ACG) have significantly lower mDNM rates. P-values and coefficients were extracted from R Poisson regression models generated with the glm function.

Motif length (bp)	All microsatellites	Microsatellites in coding exons	Non polymorphic STRs in coding exons
1	50.0%	1.8%	0.003%
2	16.7%	0.5%	0.6%
3	5.8%	76.2%	17.5%
4	13.2%	3.0%	6.4%
5	7.1%	1.5%	22.1%
6	6.5%	17.1%	53.4%

Supplementary Table 29 | **Motif length composition in exons.** Motif length composition for all microsatellites and exon intersecting microsatellites.

Motif length	All microsatellites	Coding microsatellites	Non-polymorphic STRs	Coding non-polymorphic STRs
1	0.98	0.97 (0.25)	0.97	0.95 (0.05)
2	0.95	0.96 (0.14)	0.93	0.97 ($5.9 \cdot 10^{-12}$)
3	0.93	0.89 ($9.0 \cdot 10^{-66}$)	0.94	0.93 ($1.3 \cdot 10^{-8}$)
4	0.89	0.88 (0.87)	0.93	0.96 ($5.7 \cdot 10^{-43}$)
5	0.84	0.89 ($3.0 \cdot 10^{-4}$)	0.96	0.97 ($2.3 \cdot 10^{-26}$)
6	0.81	0.80 (0.12)	0.94	0.94 ($3.8 \cdot 10^{-4}$)
Overall	0.94	0.87 ($1 \cdot 10^{-153}$)	0.95	0.95 ($9.6 \cdot 10^{-14}$)

Supplementary Table 30 | **Repeat purity values within and outside of exons.** Average purity values per motif length for all microsatellites, coding microsatellites, all non-polymorphic STRs and coding non-polymorphic STRs. Mann-Whitney U-test p-values for significant difference in purity values between exonic and non-exonic microsatellites in each motif length in brackets.

Length threshold (bp)	percentage adding bp below	percentage adding bp above
20	59.4%	52.1%
30	59.6%	50.7%
40	56.9%	49.5%
50	54.2%	49.9%
60	53.5%	51.2%
70	53.5%	50.2%
80	53.4%	46.2%
90	53.4%	50.0%
100	53.4%	42.9%

Supplementary Table 31 | **Fraction of mDNMs adding bp below and above RRT length thresholds.** Fraction of mDNMs adding bp above and below different repeat tract length thresholds. The fraction of mDNMs adding bp is higher below the threshold in all cases.

Bp	1 bp motif	2 bp motif	3 bp motif	4 bp motif	5 bp motif	6 bp motif
11-20	0.14($2.2 \cdot 10^{-73}$)	0.04($5.9 \cdot 10^{-6}$)	0.4(0.01)	0.02(0.01)	-0.005(0.39)	-0.001(0.96)
21-30	0.21($< 1.0 \cdot 10^{-320}$)	0.16($3.2 \cdot 10^{-280}$)	0.13($2.2 \cdot 10^{-31}$)	0.05($1.9 \cdot 10^{-29}$)	0.002(0.64)	0.003(0.57)
31-40	0.20($4.5 \cdot 10^{-36}$)	0.17($< 1.0 \cdot 10^{-320}$)	0.17($3.2 \cdot 10^{-118}$)	0.11($5.8 \cdot 10^{-187}$)	0.06($6.0 \cdot 10^{-24}$)	0.01(0.05)
41-50	0.12(0.01)	0.16($< 1.0 \cdot 10^{-320}$)	0.12($4.5 \cdot 10^{-89}$)	0.09($8.6 \cdot 10^{-267}$)	0.08($4.3 \cdot 10^{-35}$)	0.04($5.0 \cdot 10^{-7}$)
51-60	-0.50($3.9 \cdot 10^{-4}$)	0.15($8.5 \cdot 10^{-239}$)	0.11($7.5 \cdot 10^{-40}$)	0.07($1.4 \cdot 10^{-201}$)	0.06($2.8 \cdot 10^{-22}$)	0.06($3.2 \cdot 10^{-14}$)
61-70	-1.27(0.01)	0.13($3.6 \cdot 10^{-35}$)	0.12($3.2 \cdot 10^{-12}$)	0.06($6.0 \cdot 10^{-104}$)	0.06($2.6 \cdot 10^{-17}$)	0.05($1.5 \cdot 10^{-8}$)
71-80	0.65(0.48)	0.14($3.5 \cdot 10^{-9}$)	0.03(0.37)	0.05($2.3 \cdot 10^{-41}$)	0.03($1.5 \cdot 10^{-5}$)	0.01(0.15)
81-90	X	0.05(0.58)	0.47(0.31)	0.04($1.6 \cdot 10^{-11}$)	0.05($4.9 \cdot 10^{-5}$)	0.05(0.06)
>90	X	X	5.43(1.00)	0.03($6.0 \cdot 10^{-4}$)	0.001(0.98)	0.03(0.59)

Supplementary Table 32 | **Regression effects of purity on mDNM rate per RRT length bin and motif length.** Poisson regression coefficients and p-values for effect of repeat purity on mDNM rate split into ten bp RRT length bins stratified on motif length. Data for regression was not available for homopolymers above 80 bp and dinucleotide microsatellites above 90 bp.

Supplementary References

1. Harris, A. M. & DeGiorgio, M. An unbiased estimator of gene diversity with improved variance for samples containing related and inbred individuals of any ploidy. *G3 Genes, Genomes, Genet.* **7**, (2017).

2. Arcot, S. S., Wang, Z., Weber, J. L., Deininger, P. L. & Batzer, M. A. Alu repeats: A source for the genesis of primate microsatellites. *Genomics* **29**, (1995).

3. Grandi, F. & An, W. Non-LTR retrotransposons and microsatellites: Partners in genomic variation. *Mob. Genet. Elements* **3**, (2013).

4. Robertson, K. D. & Jones, P. A. DNA methylation: Past, present and future directions. *Carcinogenesis* **21**, (2000).

5. Buschiazzo, E. & Gemmell, N. J. The rise, fall and renaissance of microsatellites in eukaryotic genomes. *BioEssays* **28**, (2006).

6. Ellegren, H. Microsatellites: Simple sequences with complex evolution. *Nature Reviews Genetics* **5**, (2004).

7. Legendre, M., Pochet, N., Pak, T. & Verstrepen, K. J. Sequence-based estimation of minisatellite and microsatellite repeat variability. *Genome Res.* **17**, (2007).

8. Sainudiin, R., Durrett, R. T., Aquadro, C. F. & Nielsen, R. Microsatellite mutation models: Insights from a comparison of humans and chimpanzees. *Genetics* **168**, (2004).

9. Eckert, K. A. & Hile, S. E. Every microsatellite is different: Intrinsic DNA features dictate mutagenesis of common microsatellites present in the human genome. *Mol. Carcinog.* **48**, (2009).

10. Lal, A. *et al.* Improving long-read consensus sequencing accuracy with deep learning. *bioRxiv* (2021).

11. Jonsson, H. *et al.* Differences between germline genomes of monozygotic twins. *Nat. Genet.* **53**, (2021).

12. Mitra, I. *et al.* Patterns of de novo tandem repeat mutations and their role in autism. *Nature* **589**, (2021).

13. Sun, J. X. *et al.* A direct characterization of human mutation based on microsatellites. *Nat. Genet.* (2012). doi:10.1038/ng.2398

14. Udupa, S. M. & Baum, M. High mutation rate and mutational bias at (TAA)n microsatellite loci in chickpea (Cicer arietinum L.). *Mol. Genet. Genomics* **265**, (2001).

15. Kruglyak, S., Durrett, R., Schug, M. D. & Aquadro, C. F. Distribution and abundance of microsatellites in the yeast genome can be explained by a balance between slippage events and point mutations. *Mol. Biol. Evol.* **17**, (2000).

16. Murat, P., Guilbaud, G. & Sale, J. E. DNA polymerase stalling at structured DNA constrains the expansion of short tandem repeats. *Genome Biol.* **21**, (2020).

17. Khristich, A. N. & Mirkin, S. M. On the wrong DNA track: Molecular mechanisms of repeat-mediated genome instability. *Journal of Biological Chemistry* **295**, (2020).

18. Supek, F. & Lehner, B. Differential DNA mismatch repair underlies mutation rate variation across the human genome. *Nature* **521**, (2015).

19. Koren, A. *et al.* Differential relationship of DNA replication timing to different forms of human mutation and variation. *Am. J. Hum. Genet.* **91**, (2012).

20. Fan, H. & Chu, J. Y. A Brief Review of Short Tandem Repeat Mutation. *Genomics. Proteomics Bioinformatics* **5**, 7–14 (2007).

21. Lai, Y. & Sun, F. The Relationship between Microsatellite Slippage Mutation Rate and the Number of Repeat Units. *Mol. Biol. Evol.* **20**, (2003).

22. Brinkmann, B., Klintschar, M., Neuhuber, F., Hühne, J. & Rolf, B. Mutation Rate in Human Microsatellites: Influence of the Structure and Length of the Tandem Repeat. *Am. J. Hum. Genet.* (2002). doi:10.1086/301869

23. Ellegren, H. Heterogeneous mutation processes in human microsatellite DNA sequences. *Nat. Genet.* **24**, 400–402 (2000).

24. Xu, X., Peng, M., Fang, Z. & Xu, X. The direction of microsatellite mutations is dependent upon allele length. *Nat. Genet.* **24**, (2000).

25. Huang, Q. Y. *et al.* Mutation patterns at dinucleotide microsatellite loci in humans. *Am. J. Hum. Genet.* **70**, (2002).

26. Gardner, M. G., Bull, C. M., Cooper, S. J. B. & Duffield, G. A. Microsatellite mutations in litters of the Australian lizard Egernia stokesii. *J. Evol. Biol.* **13**, (2000).

27. Jones, A. G., Rosenqvist, G., Berglund, A. & Avise, J. C. Clustered microsatellite mutations in the pipefish Syngnathus typhle. *Genetics* **152**, (1999).

28. Primmer, C. R., Saino, N., Møller, A. P. & Ellegren, H. Unraveling the processes of microsatellite evolution through analysis of germ line mutations in barn swallows Hirundo rustica. *Mol. Biol. Evol.* **15**, (1998).

29. Harr, B. & Schlötterer, C. Long microsatellite alleles in Drosaphila melanogaster have a downward mutation bias and short persistence times, which cause their genome-wide underrepresentation. *Genetics* **155**, (2000).

30. Drotschmann, K., Clark, A. B. & Kunkel, T. A. Mutator phenotypes of common polymorphisms and missense mutations in MSH2. *Curr. Biol.* **9**, (1999).

31. Ellison, A. R., Lofing, J. & Bitter, G. A. Functional analysis of human MLH1 and MSH2 missense variants and hybrid human-yeast MLH1 proteins in Saccharomyces cerevisiae. *Hum. Mol. Genet.* **10**, (2001).

32. Alexandrov, L. B. *et al.* The repertoire of mutational signatures in human cancer. *Nature* **578**, (2020).
