# Supplementary material for: Sequence variants affecting the genome-wide rate of germline microsatellite mutations
Source: Nat Commun. 2023 Jun 29;14:3855. doi: 10.1038/s41467-023-39547-6 (PMC10310707; doi:10.1038/s41467-023-39547-6)
Supplement: Supplementary file 3 — Description of Additional Supplementary Files [file 41467_2023_39547_MOESM3_ESM.pdf]

**File name: Supplementary Data 1**

**Description:** Number of coding variants within mismatch repair, NER and BER genes which have GWAS p-values < 0.05 to number of transmitted mDNMs and the total number of coding variants within these genes. The counts were done for six phenotypes: all parents, fathers only and mothers only both including and excluding homopolymers. For each phenotype the binomial test (probability of success = 0.05) p-value of association enrichment in the gene is in the next column after.
