# Supplementary material for: Sequence variants affecting the genome-wide rate of germline microsatellite mutations
Source: Nat Commun. 2023 Jun 29;14:3855. doi: 10.1038/s41467-023-39547-6 (PMC10310707; doi:10.1038/s41467-023-39547-6)
Supplement: Supplementary file 5 — Reporting Summary [file 41467_2023_39547_MOESM5_ESM.pdf]

nature portfolio

Corresponding author(s): Bjarni V.Halldorsson
Kari Stefansson

Last updated by author(s): Jun 5, 2023

Reporting Summary

Nature Portfolio wishes to improve the reproducibility of the work that we publish. This form provides structure for consistency and transparency in reporting. For further information on Nature Portfolio policies, see our Editorial Policies and the Editorial Policy Checklist.

Statistics

For all statistical analyses, confirm that the following items are present in the figure legend, table legend, main text, or Methods section.

n/a	Confirmed	
☐	☒	The exact sample size (*n*) for each experimental group/condition, given as a discrete number and unit of measurement
☐	☒	A statement on whether measurements were taken from distinct samples or whether the same sample was measured repeatedly
☐	☒	The statistical test(s) used AND whether they are one- or two-sided<br>*Only common tests should be described solely by name; describe more complex techniques in the Methods section.*
☐	☒	A description of all covariates tested
☐	☒	A description of any assumptions or corrections, such as tests of normality and adjustment for multiple comparisons
☐	☒	A full description of the statistical parameters including central tendency (e.g. means) or other basic estimates (e.g. regression coefficient) AND variation (e.g. standard deviation) or associated estimates of uncertainty (e.g. confidence intervals)
☐	☒	For null hypothesis testing, the test statistic (e.g. *F*, *t*, *r*) with confidence intervals, effect sizes, degrees of freedom and *P* value noted<br>*Give P values as exact values whenever suitable.*
☒	☐	For Bayesian analysis, information on the choice of priors and Markov chain Monte Carlo settings
☒	☐	For hierarchical and complex designs, identification of the appropriate level for tests and full reporting of outcomes
☐	☒	Estimates of effect sizes (e.g. Cohen's *d*, Pearson's *r*), indicating how they were calculated

Our web collection on statistics for biologists contains articles on many of the points above.

Software and code

Policy information about availability of computer code

Data collection	We used popSTR v2.0(https://github.com/DecodeGenetics/popSTR) to generate microsatellite genotypes. To analyze the mDNMs we used scripts available here: https://github.com/DecodeGenetics/mDNM_analysisAndData.
Data analysis	We used R (v.3.6.3) https://www.r-project.org/ and Python (v.2.7.5) to analyze data and create plots. To generate confidence intervals we used version 1.3-28 of the boot package for R.

For manuscripts utilizing custom algorithms or software that are central to the research but not yet described in published literature, software must be made available to editors and reviewers. We strongly encourage code deposition in a community repository (e.g. GitHub). See the Nature Portfolio guidelines for submitting code & software for further information.

Data

Policy information about availability of data

All manuscripts must include a data availability statement. This statement should provide the following information, where applicable:

- Accession codes, unique identifiers, or web links for publicly available datasets
- A description of any restrictions on data availability
- For clinical datasets or third party data, please ensure that the statement adheres to our policy

Access to these data is controlled; the sequence data cannot be made publicly available because Icelandic law and the regulations of the Icelandic Data Protection Authority prohibit the release of individual-level and personally identifying data. Data access can be granted only at the facilities of deCODE genetics in Iceland,

subject to Icelandic law regarding data usage. Anyone wishing to gain access to the data should contact K.S. (kstefans@decode.is) with a timeframe of one month for a response. Results from the association analysis to our phenotypes will be uploaded to the deCODE genetics website www.decode.com/summarydata. A list of the mDNMs generated and used in the study is available here: https://doi.org/10.5281/zenodo.8005262. We have also uploaded a list of the markers considered and mDNM counts per marker to the same github repository80. WGS, genotype data, phased and imputed data for the UKB set can be accessed via the UKB research analysis platform (RAP): https://ukbiobank.dnanexus.com/landing. The Research Analysis Platform is open to researchers who are listed as collaborators on UKB-approved access applications. The UKB microsatellite genotypes were created as a part of this study and also presented in https://doi.org/10.1038/s41586-022-04965-x. This research has been conducted using the UK Biobank Resource under Application number 68574.

Human research participants

Policy information about studies involving human research participants and Sex and Gender in Research.

Reporting on sex and gender	We study sex and not gender, consequently only the term "sex" is used in the study. All analysis is done jointly for the two sexes and split by sex.
Population characteristics	Characteristics of the UK biobank have been described in Sudlow, C. et al. UK Biobank: An Open Access Resource for identifying the Causes of a Wide Range of Complex Diseases of Middle and Old Age. PLOS Med. 12, e1001779 (2015). The description of the data acquisition and processing of the Icelandic set is described in Jónsson, H., Sulem, P., Kehr, B. et al. Whole genome characterization of sequence diversity of 15,220 Icelanders. Sci Data 4, 170115 (2017).
Recruitment	All participants were Icelanders who donated biological samples to explore the interplay between the genetic variation and phenotypic diversity, and were provided informed consents as part of various genetic programs at deCODE genetics, Reykjavík, Iceland. The sample set may be enriched for families/close-relatives and cases compared to controls, however it should be sufficiently large to represent the Icelandic population. Recruitment of individuals to the UK biobank has been described in previous studies.
Ethics oversight	For Icelandic samples: The National Bioethics Committee and the Icelandic Data Protection Authority approved this study. Blood or buccal samples were taken from individuals participating in various studies, after receiving informed consent from them or their guardians.<br>The North West Research Ethics committee reviewed and approved the UKB's scientific protocol and operational procedures (REC Reference number: 06/MRE08/65). Data for this study were obtained and research conducted under the UKB applications license number 68574

Note that full information on the approval of the study protocol must also be provided in the manuscript.

Field-specific reporting

Please select the one below that is the best fit for your research. If you are not sure, read the appropriate sections before making your selection.

☒ Life sciences ☐ Behavioural & social sciences ☐ Ecological, evolutionary & environmental sciences

For a reference copy of the document with all sections, see nature.com/documents/nr-reporting-summary-flat.pdf

Life sciences study design

All studies must disclose on these points even when the disclosure is negative.

Sample size	The UKB has approximately 500,000 samples, a pseudo-random subset of 150,119 of those were sequenced. No statistical analysis was performed to choose sample size. The choice of sample size was a balance between purported power in association analysis and a consideration of the time from start and finish of the project. The Icelandic set has 53,026 samples chosen to not have polymerase chain reaction (PCR) as a part of their sample preparation library which were all such WGS samples available at deCODE at the beginning of the study. We believe these samples sizes are sufficient since these are two of the largest microsatellite genoytpe sets reported on till date.
Data exclusions	A small subset of individuals from the UKB withdrew consent during the time of study and samples with PCR as a part of their sample preparation library were excluded from the Icelandic set.
Replication	Our results on the effects of microsatellite attributes on polymorphism rates and expected heterozygosity values were replicated between the two sets. Our mDNM results from the Icelandic set were not replicated in this study since the UKB set did not contain a large enough number of parent offspring trios and we did not have access to another set of parent offspring trios of a sufficient size. Many of the mutational patterns we report however replicate previous reports.
Randomization	Samples were pseudorandomly selected among the 500,000 samples in the UK biobank and the pseudorandomly distributed between the two sequencing centers. Not relevant for Icelandic samples as there were no group allocations of samples.
Blinding	Investigators were blinded to the randomization of the UK biobank samples and there were no group allocations of samples in the Icelandic set.

Reporting for specific materials, systems and methods

We require information from authors about some types of materials, experimental systems and methods used in many studies. Here, indicate whether each material, system or method listed is relevant to your study. If you are not sure if a list item applies to your research, read the appropriate section before selecting a response.

Materials & experimental systems

n/a	Involved in the study
☒	☐ Antibodies
☒	☐ Eukaryotic cell lines
☒	☐ Palaeontology and archaeology
☒	☐ Animals and other organisms
☒	☐ Clinical data
☒	☐ Dual use research of concern

Methods

n/a	Involved in the study
☒	☐ ChIP-seq
☒	☐ Flow cytometry
☒	☐ MRI-based neuroimaging
